# The NEuroCOUGH Chronic Cough Registry: a protocol for a pan-European observational study

**DOI:** 10.1183/23120541.00289-2025

**Published:** 2025-09-22

**Authors:** Peter S.P. Cho, Surinder S. Birring, Clíona McDowell, Tony Brown, James H. Hull, Jaclyn A. Smith, Laurent Guilleminault, Peter Kardos, Jan W. van den Berg, Christian Domingo, Paul Marsden, Marco Idzko, Federico Lavorini, Matthew J. Martin, Claire Slinger, Silvia Demoulin-Alexikova, Daiana Stolz, Arent Jan Michels, Madara Tirzite, Brigita Gradauskiene, Joao Carlos Winck, Ossur Ingi Emilsson, Georgios Kaltsakas, Charlotte Hyldgaard, Kian Fan Chung, Eva Millqvist, Ajit Narayanan, Lisa Gossage, Khan Buchwald-Mackintosh, Richard J. Siegert, Marianne Schulte, Abitha Nair, Jemma Nelson, Sean M. Parker, Marta Dąbrowska, Alyn Morice, Lieven J.A. Dupont, Lorcan McGarvey

**Affiliations:** 1 Department of Respiratory Medicine, King's College Hospital NHS Foundation Trust, London, UK; 2 Centre for Human and Applied Physiological Sciences, King's College London, London, UK; 3 Northern Ireland Clinical Research Network, Belfast, UK; 4 Airway Disease Section, Royal Brompton Hospital, Guy's and St Thomas’ NHS Foundation Trust, London, UK; 5 University College London, London, UK; 6 NIHR Manchester Clinical Research Facility, School of Biological Sciences, Faculty of Biology, Medicine and Health Sciences, The University of Manchester, Manchester, UK; 7 Manchester University NHS Foundation Trust, Manchester, UK; 8 Toulouse Institute for Infectious and Inflammatory Diseases (Infinity), INSERM UMR1291, CNRS UMR5051, University Toulouse, Toulouse, France; 9 Department of Respiratory Medicine, Toulouse University Hospital, Faculty of Medicine, Toulouse, France; 10 Group Practice and Centre for Allergy, Respiratory and Sleep Medicine, Red Cross Maingau Hospital, Frankfurt, Germany; 11 Isala Hospital, Zwolle, Netherlands; 12 Pulmonology Department, Consorci Corporació Sanitària Parc Taulí, Barcelona, Spain; 13 Department of Medicine, Universitat Autònoma de Barcelona, Barcelona, Spain; 14 Department of Pneumology, University Hospital Vienna AKH, Medical University of Vienna, Vienna, Austria; 15 Department of Experimental and Clinical Medicine, University of Florence, Florence, Italy; 16 Nottingham Respiratory Research Unit, Nottingham Biomedical Research Centre, Nottingham University Hospitals NHS Trust, Nottingham, UK; 17 Lancashire Teaching Hospitals NHS Trust, Preston, UK; 18 Univ. Lille, CNRS, Inserm, CHU Lille, Institut Pasteur de Lille, U1019 – UMR 9017 – CIIL – Center for Infection and Immunity of Lille, Lille, France; 19 Clinic of Pneumology, Medical Center-University of Freiburg, Faculty of Medicine, University of Freiburg, Freiburg, Germany; 20 Anna Hospital, Geldrop, Netherlands; 21 Riga Stradins University, Riga, Latvia; 22 Clinical centre Gailezers, Riga East University Hospital, Riga, Latvia; 23 Department of Immunology and Allergology, Lithuanian University of Health Sciences, Kaunas, Lithuania; 24 Cardiovascular R&D Centre, Faculdade de Medicina da Universidade do Porto, Porto, Portgual; 25 Chronic Cough Clinic and Sleep and Ventilation Unit, Instituto CUF Porto, Porto, Portugal; 26 Department of Medical Sciences, Respiratory, Allergy and Sleep Research, Uppsala University, Uppsala, Sweden; 27 Faculty of Medicine, University of Iceland, Reykjavik, Iceland; 28 University Department of Respiratory Medicine, National and Kapodistrian University of Athens, “Sotiria” Chest Hospital, Athens, Greece; 29 Lane Fox Respiratory Service, Guy's and St Thomas’ NHS Foundation Trust, London, UK; 30 Medical Diagnostic Center, University Clinic for Innovative Patient Pathways, Regional Hospital Central Jutland, Viborg, Denmark; 31 National Heart and Lung Institute, Imperial College London, London, UK; 32 Department of Internal Medicine/Respiratory Medicine and Allergology, Sahlgrenska University Hospital, University of Gothenburg, Gothenburg, Sweden; 33 Department of Data Science and AI, Auckland University of Technology, Auckland, New Zealand; 34 Department of Psychology and Neuroscience, Faculty of Health and Environmental Sciences, Auckland University of Technology, Auckland, New Zealand; 35 School of Clinical Sciences, Auckland University of Technology, Auckland, New Zealand; 36 UZ Leuven, Leuven, Belgium; 37 Belfast City Hospital, Belfast, UK; 38 Northumbria Healthcare NHS Foundation Trust, North Shields, UK; 39 Department of Respiratory Medicine, North Tyneside General Hospital, Northumbria Healthcare NHS Foundation Trust, North Shields, UK; 40 Department of Internal Medicine, Pulmonary Diseases and Allergy, Medical University of Warsaw, Warsaw, Poland; 41 Hull York Medical School, University of Hull, Castle Hill Hospital, Cottingham, UK; 42 Department of Respiratory Diseases, University Hospital Leuven, Katholieke Universiteit Leuven, Leuven, Belgium; 43 Wellcome Wolfson Institute of Experimental Medicine, School of Medicine, Dentistry and Biomedical Sciences, Queen's University Belfast, Belfast, UK

## Abstract

Chronic cough is a common clinical problem which is burdensome for patients and can be difficult to treat. Individual centres of cough expertise have been set up in countries across Europe but to date there has been no means to collate and analyse clinical data from such sites to gain a more comprehensive understanding of cough phenotypes, disease burden and the natural history of this condition. The NEw Understanding in the tReatment Of COUGH (NEuroCOUGH) registry is the first pan-European prospective observational study of adult patients referred for evaluation of chronic cough. Patients (≥18 years old) with a cough lasting more than 8 weeks with no chest radiology findings to explain the cough will be recruited. Key exclusion criteria include current or recent (<12 months) smoking, significant smoking history (≥20 pack-years) and obstructive spirometry (forced expiratory volume in 1 s/forced vital capacity ratio <0.6). The study aims to recruit 2500 patients across 13 European sites by 2026 and participants will be followed up at 12-monthly intervals for 36 months. The registry comprises comprehensive clinical, physiological and biological data on chronic cough, along with data on the impact and longitudinal outcome of chronic cough. The NEuroCOUGH registry has been established at a time of considerable advance in the field of cough. It will serve as a valuable clinical and research resource which will extend our current understanding of this difficult to treat condition. The initiative is also intended to encourage the establishment of new specialist cough clinics, thus creating much needed clinical trial infrastructure throughout Europe to ultimately improve patient care.

## Shareable abstract

**The ERS NEuroCOUGH CRC Chronic Cough Registry is the first multicentre European observational study comprising clinical, physiological, biological and outcome data to provide insight on phenotypes and endotypes of chronic cough to improve patient care** https://bit.ly/3ElhVi1

## Introduction

Chronic cough is a common clinical problem which can be difficult to treat especially when therapy directed at common pulmonary and extrapulmonary causes is ineffective. Patients with chronic cough typically report the problem as one persisting for many years requiring repeated healthcare visits and numerous negative investigations, often with little or no response to medication, leaving them frustrated and generally dissatisfied with their treatment journey [1, 2]. The recognition that clinical management of chronic cough needed to improve prompted the development of international consensus on its evaluation and treatment [3, 4]. A welcome consequence of this initiative was the establishment of specialist cough clinics in a number of countries around the world [5]. The clinical experience from these centres suggests that distinct demographic and clinical patterns or phenotypes commonly exist [6]. To date, no cross-sectional or longitudinal studies of multinational chronic cough patient cohorts have been undertaken, undoubtedly due to the high level of multidisciplinary cooperation needed to undertake such studies. To that end, in 2018, the NEw Understanding in the tReatment Of COUGH (NEuroCOUGH) Clinical Research Collaboration (CRC) was established as part of the European Respiratory Society (ERS) initiative to support the coordination of activities in respiratory medicine between multiple stakeholders and centres across Europe (www.ersnet.org/science-and-research/clinical-research-collaboration-application-programme/) [7].

A primary objective of the ERS NEuroCOUGH CRC was to establish a network of clinics across Europe and beyond to encourage the evaluation and management of chronic cough patients in an agreed and standardised manner internationally [4, 7]. A further priority was the creation of a Europe-wide registry of well-characterised patients with chronic cough for recruitment to multicentre clinical studies of novel antitussive therapies. Here, we describe the protocol of the NEuroCOUGH Chronic Cough Registry, which had the following objectives: to establish the first pan-Europe multicentre chronic cough registry with clinical, physiological and biological data collected at baseline and annual follow-up for 36 months; to describe demographics, cough characteristics, co-morbidities, aetiologies, management and impact of chronic cough, and its various phenotypes and endotypes across Europe; to encourage and facilitate the establishment of clinical trial infrastructure for cough in European countries where such has not yet been established; and to foster and maintain strong multicentre clinical and research collaboration in the field of chronic cough.

## Methods

### Study design

The NEuroCOUGH Chronic Cough Registry is a multicentre, prospective, observational cohort study designed to enrol adult patients with chronic cough referred to secondary and tertiary centres with a specialist interest in chronic cough across Europe. Comprehensive data across domains of demographics, anthropometrics, co-morbidities, cough characteristics, aetiologies, investigations, treatment trials and impact of chronic cough will be collected at baseline (recruitment) with a more focused data set at yearly follow-up.

The study is sponsored by Queen's University Belfast, Belfast, Northern Ireland and received ethics approval from the Multi-centre Research Ethics Committee in the UK on 26 July 2021 (20/EE/0213) with local ethics approval for each European site obtained by the relevant principal investigator. The study uniform resource locator is https://europeanlung.org/neurocough/.

### Participants

Patients should have a primary problem of chronic cough and meet the following inclusion and exclusion criteria. The inclusion criteria are:

- adult (≥18 years of age);
- chronic cough of >8 weeks in duration; and
- no chest radiology findings suggestive of pathology causing chronic cough.

The exclusion criteria are:

- current smoker or smoking within last 12 months;
- cumulative smoking history of ≥20 pack-years;
- forced expiratory volume in 1 s (FEV_1_) to forced vital capacity (FVC) ratio <0.60;
- acute respiratory tract infection within 4 weeks of baseline; and
- patients who are unable or unwilling to provide informed consent.

### Recruitment and follow-up

**FIGURE 1:**
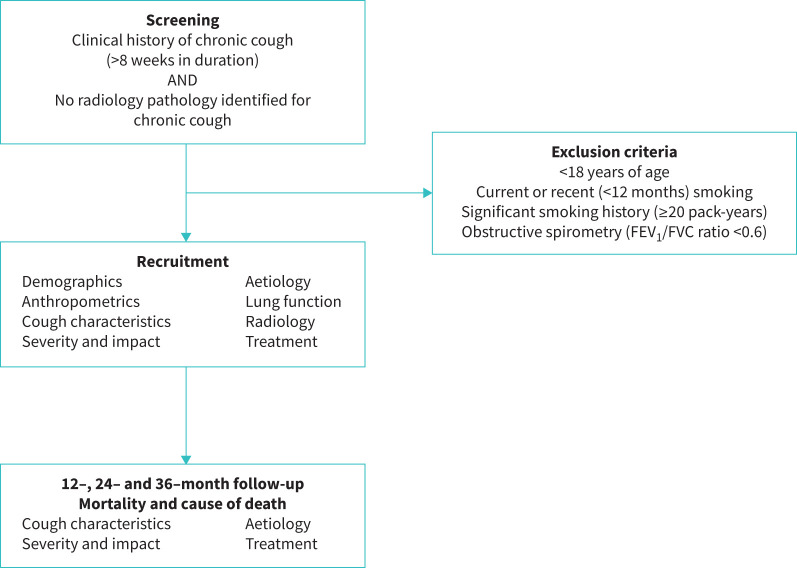
Study flowchart. FEV_1_: forced expiratory volume in 1 s; FVC: forced vital capacity.

Patients with chronic cough will be identified from those referred to clinics in secondary or tertiary care settings with an interest in chronic cough. Patients will be managed by the responsible clinical teams according to the local procedures and policies. Following completion of baseline data collection, all participants will be invited to enter annual (±3 months) follow-up over the following 36 months (figure 1 and supplementary table E1). Longitudinal data on cough characteristics, investigations, treatment trials, aetiology, severity, complications and impact of cough, and mortality will be collected (supplementary table E2).

### Data collection and entry

Data will be collected at distinct time points: baseline (data obtained at study enrolment) and annual review at 12-, 24- and 36-month follow-up where data is obtained at scheduled “in-person” clinic visits or alternatively a phone call/virtual clinic follow-up can be undertaken. Data will be entered through a collection platform supported by University of Dundee Health Informatics Centre (HIC) (https://hicsesrvices.staging.dundee.ac.uk/neurocough).

**FIGURE 2:**
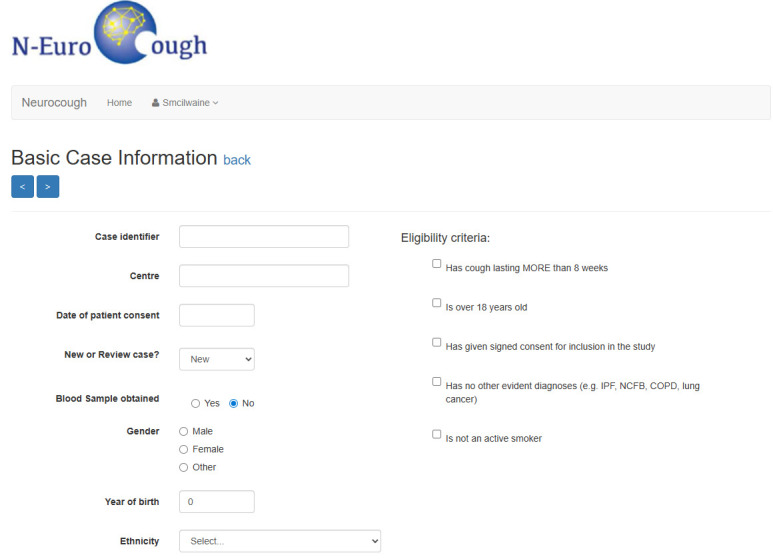
NEw Understanding in the tReatment Of COUGH (NEuroCOUGH) cough registry data collection platform (https://neurocough.hicservices.dundee.ac.uk/).

The data fields were agreed following consultation with cough specialists across Europe with input from the NEuroCOUGH and European Lung Foundation (ELF) Patient Advisory Group (PAG) and are summarised in supplementary tables E1 and E2. The intent is for an extensive dataset, which will enable additional ancillary studies to be proposed by investigators. All data will be entered on an electronic case report form (figure 2). In addition, participants are invited to provide consent for future contact regarding participation in clinical trials and studies.

#### Cough characteristics, triggers and complications

Participants self-report duration of cough, cough frequency and sputum production, including volume and appearance. In addition, participants self-report any triggers of cough, relieving factors for cough and respiratory symptoms and complications associated with cough.

#### Severity and impact

Cough severity and urge to cough are self-reported on visual analogue scales (VAS) (range: 0–100 mm) and modified Borg (mBorg) scales (range: 0–10) (in the VAS and mBorg scales, higher scores indicate more severe cough or worse urge, respectively) [8–10]. Cough-specific health status is assessed with the self-administered 19-item Leicester Cough Questionnaire, which is validated in chronic cough and widely translated (range: 3–21; higher scores indicate better health status) [11, 12]. Generic health status is assessed with the EuroQol EQ-5D-5L and responses will be converted to a numeric score (range: 0–1; one indicates full health, zero indicates death and below zero indicates a health status considered worse than death) [13].

#### Aetiology

The aetiology of chronic cough is determined by the clinician responsible for the patient based on their interpretation of investigational data and outcome of treatment trials in line with consensus statements [3, 14]. The following categories are included with the option to include multiple aetiologies and provision for free text should additional aetiological descriptors be required: refractory chronic cough; unexplained chronic cough; post-infective; classic asthma; cough variant asthma; eosinophilic bronchitis; gastroesophageal reflux disease; upper airway cough syndrome; aspiration; and not yet determined (under further investigation).

#### Lung function

Raw values for FEV_1_, FVC, anthropometrics and ethnicity are collected to enable the appropriate calculations for predicted values [15]. Bronchodilator response, bronchial challenge testing, fractional exhaled nitric oxide and cough provocation testing are recorded where available. Raw values for body plethysmography and transfer coefficient for carbon monoxide are also collected, where available. Spirometry, body plethysmography and transfer coefficient testing are all performed as per the recommendations of the American Thoracic Society/ERS guidelines [16].

#### Radiology

The results of the most recent chest radiology (chest radiograph and/or chest computed tomography (CT)), preferably within 2 years, will be recorded. Although study inclusion criteria require the absence of any chest radiology findings that could potentially explain chronic cough, provision is made for free text to record co-existent radiological features considered unrelated to chronic cough.

#### Blood investigations

The serum eosinophil value at baseline and the peak serum eosinophil value within the preceding 24 months are collected, whilst radioallergosorbent test results for common allergens are collected, where available.

#### Treatment trials

All pharmacotherapy and nonpharmacotherapy received to date along with therapeutic response to said treatments are recorded.

#### Genetic and transcriptomics

Participants provide blood samples (10 mL PAXgene DNA and 10 mL PAXgene RNA) for genetic testing and transcriptomics at baseline on an optional basis. All samples are stored locally (−80°C freezer) prior to transfer to central laboratories (Queen University Belfast, Belfast, Northern Ireland). Transfer and storage of material will be tracked using a sample-tracking database. All samples are stored in accordance with the Human Tissue Act 2004 for at least 15 years. Samples shall not be redistributed or released to any individuals other than for the purpose of the protocol or ancillary studies approved by an appropriate ethics committee and in accordance with the participants’ consent.

### Quality control

The database comprises an automated logic check to alert users of any out-of-range values and such values are prevented from being entered. Each case is manually verified by a member of the study team and data queries are resolved with the study site. Cases with missing core cough or demographic data that remain unresolved despite efforts of quality control team will be excluded from analysis. Each study site may be subjected to random inspection and audit for data verification and to ensure adherence to the protocol.

### Sample size and statistical analysis

**FIGURE 3:**
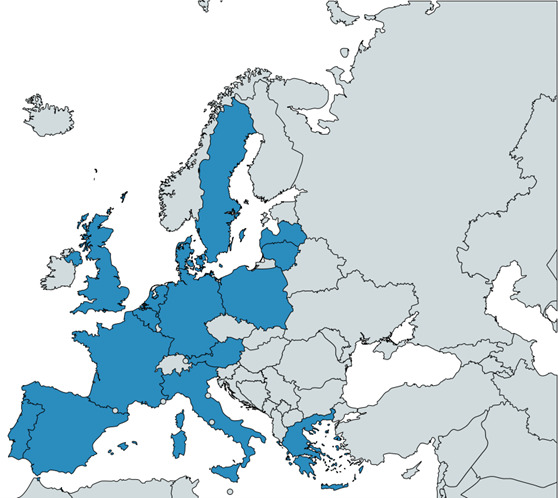
NEw Understanding in the tReatment Of COUGH (NEuroCOUGH) countries across Europe.

The sample size is empirically determined at 2500 patients across Europe and the registry has no maximum number of patients. A short-term target of 1000 patients in the first 3 years of the project was reached at the end of 2024, with patients recruited from 12 sites across seven European countries. A further 10 sites from eight additional countries will be activated in 2025 (figure 3). Taken together, we believe the registry data will be broadly representative of the characteristics and management of chronic cough patients across Europe.

Descriptive analyses of the baseline data will be carried out with baseline demographic, clinical and cough data summarised as mean, standard deviation, median, interquartile range or numbers and frequencies (%) as appropriate. This will depend on the scale of measurement and distribution broken down by refractory and unexplained chronic cough, and by gender. There will be no significance testing performed initially.

When 12-month data collection is complete, we will use paired t-tests on these questionnaires to test for changes against the baseline data. Alternatively, Wilcoxon signed-rank tests will be used depending on whether assumptions are violated in the paired t-test. When 24-month and 36-month data are complete, we will use the repeated measures ANOVA or the Friedman test based whether assumptions are violated, to compare across multiple groups.

Exploratory analysis to identify new phenotypes of chronic cough that may lie hidden in patterns of responses within and across subpopulations (*e.g.* within aetiology, between locations) will be undertaken through the application of advanced statistical and machine learning techniques, such as clustering and network analysis. Results from such techniques will be scrutinised for clinical relevance and understanding so that future observational studies can develop specific hypotheses for clinical application and possible new targets for treatments.

### Governance and data sharing

The registry is held securely in the University of Dundee HIC and anonymised data are accessible to approved investigators through the Safe Haven framework. The HIC “Safe Haven” platform is a virtual desktop (Trusted Research Environment) which allows secure data access and data analysis but prevents copying or alteration of data, thereby ensuring complete data security. The HIC services security policy is available at https://hicservices.atlassian.net/wiki/spaces/HICSOP/pages/460128257/Information+Security+Policy. Investigators and other stakeholders will have unrestricted access to their own data. Requests to analyse the database as a whole will be managed by submission of a study protocol to the NEuroCOUGH scientific committee. Requests for data access from external agencies or investigators will be discussed on a case-by-case basis by the NEuroCOUGH scientific committee. The HIC database and governance processes surrounding data management and access are fully compliant with the Data Protection Act 2018 and the Data Protection Directive 95/46/EC of the European Parliament and of the Council 1995. The full HIC standard operating procedures are listed at https://hicservices.atlassian.net/wiki/spaces/HICSOP/overview.

The study will be conducted in accordance with the principles of good clinical practice. A favourable ethical opinion will be obtained by each partner site from the appropriate research ethics committee. Additionally, any other necessary approvals required by partner sites will be obtained prior to commencement of the study at site. All patients must provide written informed consent to participate. It is the responsibility of the investigators at individual sites to obtain the appropriate approvals and to ensure that informed consent is in place. The online supplement summarises registry governance, data access and publication policy (appendix 1).

Study results will be disseminated in the format of official newsletters or reports, conference abstracts and peer-reviewed publications (https://europeanlung.org/neurocough/).

### Patient participation and involvement

An overarching principle of NEuroCOUGH CRC has been the inclusion of people with chronic cough and with support from the European Lung Foundation, a cough patient advisory group (PAG) was established (https://europeanlung.org/en/news-and-blog/meet-the-elf-cough-pag/). The PAG comprises 12 patients with chronic cough from six different European countries at present. The PAG provides input to the study design and implementation of the registry and NEuroCOUGH activities, thus ensuring NEuroCOUGH activities are patient focused. In addition, the PAG will support the dissemination of NEuroCOUGH outputs by providing patient resources on diagnosis and management, and through public awareness activities.

## Discussion

Chronic cough is an extremely common respiratory problem and yet our understanding of the clinical features and characteristics of this condition is drawn mainly from the experience of single centres or a few relatively small national patient registries [17–19]. The ERS NEuroCOUGH registry represents the first multi-national dataset comprising clinical, physiological and biological data from patients referred for management of chronic cough. It has been developed by incorporating a number of the features employed by the successful European Multicentre Bronchiectasis Audit and Research Collaboration registry [20]. This includes a minimum required dataset that would be captured ordinarily during the clinical evaluation and management of patients with chronic cough. This pragmatic approach the lessens burden on patients and participating sites. Coupled with a shared data entry platform with regular quality assurance checks, it will provide a valuable resource for advancing current understanding of chronic cough including its natural history, the range and prevalence of clinical phenotypes, and the severity and burden reported by people with chronic cough over an extended period of time. A recent preliminary descriptive analysis of almost 1000 chronic cough patients from the NEuroCOUGH registry confirmed clinical characteristics, patient demographics, comorbidities and cough burden consistent with that reported in smaller studies and comparable to the patients typically recruited to clinical trials of novel antitussives [21].

Recent clinical and mechanistic understanding of cough, including a recognition of the concept of cough hypersensitivity syndrome, has accelerated drug discovery in this field with the need of largescale multicentre clinical trials of novel cough therapies. The NEuroCOUGH registry will be well placed to provide a suitable platform to facilitate such endeavours. Indeed, to date, NEuroCOUGH sites have participated as recruiting centres for a number of phase II and III clinical trials of new cough therapies [22–26]. It is anticipated that with the recent approval by the European Medicines Agency for gefapixant for the treatment of refractory and unexplained chronic cough, and the longitudinal design of the NEuroCOUGH registry, determining the clinical and biological characteristics of responder and nonresponders to this therapy in a real-world setting will be possible [27].

The NEuroCOUGH CRC initiative also encourages investigator-initiated ancillary studies which are assessed by the Scientific Committee. To date, successful proposals include a network analysis to provide novel insight into the dependencies between phenotypes of chronic cough, studies to determine the predictive value of blood eosinophil count and the utility of chest CT scanning in the management of chronic cough, the utility and feasibility of ambulatory cough recording beyond 24 h and an international comparison of clinical characteristics between the NEuroCOUGH Registry and the Korean Chronic Cough Registry [17].

While the NEuroCOUGH registry aims to recruit patients across 20 European countries, there is an intention to establish similar registries in North America, Latin America and Asia and so provide a greater real-world perspective on this common and difficult-to-treat condition.

In conclusion, the NEuroCOUGH pan-European cough registry represents a significant advancement in the field of chronic cough by providing comprehensive insights to its prevalence, diagnostic patterns, associated conditions, and long-term progression. This multinational initiative will not only enhance our understanding of cough phenotypes but also serve as a robust platform for clinical and translational research, ultimately guiding the development of more effective diagnostic and therapeutic strategies.

## Data Availability

Further information on study protocol, and copies of the participant information sheet, informed consent form and data collection *pro forma* are available on the NEuroCOUGH website (https://europeanlung.org/neurocough/)

## Supplementary material

**Please note:** supplementary material is not edited by the Editorial Office, and is uploaded as it has been supplied by the author. Table S1 00289-2025.SUPPLEMENT Table S2 00289-2025.SUPPLEMENT2 Supplementary material 00289-2025.SUPPLEMENT3
