# Supplementary material for: The NEuroCOUGH Chronic Cough Registry: a protocol for a pan-European observational study
Source: ERJ Open Res. 2025 Sep 22;11(5):00289-2025. doi: 10.1183/23120541.00289-2025 (PMC12451592; doi:10.1183/23120541.00289-2025)
Supplement: Supplementary file 1 [file 00289-2025.SUPPLEMENT.pdf]

Table E1. Data collected by the NEw Understanding in the tReatment Of COUGH (NEuroCOUGH) cough registry at baseline

Categories	Variables
Demographics and anthropometrics	Age<br>Sex<br>BMI
Smoking history	Smoking status<br>Pack year history
Cough characteristics	Duration<br>Frequency<br>Sputum production and volume
Cough triggers[a]	Temperature change<br>Fragrance<br>Talking<br>Exercise
Co-morbidities[a]	Rhinosinusitis<br>Gastro-oesophageal disease<br>Neuralgia<br>Anxiety<br>Depression
Medications[a]	ACEi<br>Anti-histamine<br>Neuromodulator<br>Opioid
Lung function	FEV1<br>FVC<br>Bronchoprovocation[b]<br>FeNO[b]
Serum	Eosinophil

	RAST
Radiology	Chest radiograph<br>Chest computed tomography[b]
Severity and impact	VAS<br>LCQ<br>EQ-5D-5L
Aetiology[a]	Asthma<br>Chronic rhino-sinusitis<br>Gastro-oesophageal diseases<br>Refractory chronic cough<br>Other
Treatment[a]	Inhaler therapy<br>PPI<br>Nasal steroid<br>Neuromodulator<br>Opioid
Complications[a]	Syncope<br>Urinary incontinence<br>Sick leave

BMI = body mass index, EQ-5D-5L = 5-level EuroQoL, FeNO = fractional exhaled nitric oxide, LCQ = Leicester Cough Questionnaire, PPI = proton pump inhibitor, VAS = visual analogue scale

[a]Examples provided and non-exhaustive

[b]Not included in minimum required data set
