# Supplementary material for: The NEuroCOUGH Chronic Cough Registry: a protocol for a pan-European observational study
Source: ERJ Open Res. 2025 Sep 22;11(5):00289-2025. doi: 10.1183/23120541.00289-2025 (PMC12451592; doi:10.1183/23120541.00289-2025)
Supplement: Supplementary file 2 [file 00289-2025.SUPPLEMENT2.pdf]

**Table E2.** Data fields of the NEw Understanding in the tReatment Of COUGH (NEuroCOUGH) cough registry at annual follow up

| Categories | Variables |
|---|---|
| Mortality | Date of death<br>Cause of death |
| Cough characteristics | Frequency<br>Sputum production and volume |
| Severity and impact | VAS<br>LCQ<br>EQ-5D-5L |
| Aetiology[a] | Asthma<br>Chronic rhino-sinusitis<br>Gastro-oesophageal diseases<br>Refractory chronic cough<br>Other |
| Treatment[a] | Inhaler therapy<br>PPI<br>Nasal steroid<br>Neuromodulator<br>Opioid |
| Complications[a] | Syncope<br>Urinary incontinence<br>Sick leave |

EQ-5D-5L = 5-level EuroQoL, FEV1 = forced expiratory volume in 1 second, FVC = forced vital capacity, LCQ = Leicester Cough Questionnaire, PPI = proton pump inhibitor, VAS = visual analogue scale

[a]Examples provided and non-exhaustive
