# Supplementary material for: The NEuroCOUGH Chronic Cough Registry: a protocol for a pan-European observational study
Source: ERJ Open Res. 2025 Sep 22;11(5):00289-2025. doi: 10.1183/23120541.00289-2025 (PMC12451592; doi:10.1183/23120541.00289-2025)
Supplement: Supplementary file 3 [file 00289-2025.SUPPLEMENT3.pdf]

Appendix 1. Registry governance, data access and publication policy

REGISTRY GOVERNANCE ARRANGEMENTS

NEuroCOUGH is composed of a Steering Committee comprising the Co-chairs, 3 members of the Committee of National Leads (rotating on an annual basis), a member of the Patient Advisory Group (PAG) and the Early Career Member responsible for the oversight of the CRC as well as for the reporting to ERS. A NEuroCOUGH Registry Scientific Committee responsible for the running of the registry will be developed. The Registry Scientific Committee will have direct responsibility for the conduct of the registry including ensuring compliance with the protocol. The Registry Scientific Committee will work with the Steering Committee to provide direction on the strategic development of the Registry. The Registry Scientific Committee will have the primary role for screening applications for access to registry data and the monitoring of progress with projects.

MEMBERSHIP AND ROLES

The membership of the Registry Scientific Committee comprises a maximum of 7 members elected from the Steering Committee and Committee of National Leads and must include a member of the PAG. The Chair of the Registry Scientific Committee will be determined by a vote of members of the Registry Scientific Committee. Appointments to the Registry Scientific Committee are for a period of 2 years, renewable once.

There must be >50% of the Registry Scientific Committee members present at a meeting in order for decisions to be taken. A majority decision is taken in all cases. In the event of a tied vote the agenda item will be deferred to the next meeting and re-presented. Two episodes of tied votes indicates there is not a majority and the proposal will be declined.

Agenda Items and Papers

The Registry Scientific Committee agenda, with attached meeting papers will be distributed at least 30 days prior to the next scheduled meeting.

The Chair has the right to decline to list an item on the formal agenda, but members may raise an item under “Other Business” if necessary and as time permits.

Full copies of the Minutes, including attachments, shall be provided to all Steering Committee members no later than 30 working days following each meeting.

By agreement of the Committee, out-of-session decisions will be deemed acceptable. Where agreed, all out-of-session decisions shall be recorded in the minutes of the next scheduled Steering Committee meeting.

Frequency of Meetings

The Chair shall convene Registry Scientific Committee meetings at a minimum frequency of twice yearly with teleconferences as required throughout the year. If more than 4 proposals are expected at a steering committee meeting the Chair will call an additional meeting. The meetings should be aligned where possible with the NEuroCOUGH general meetings or the ERS annual congress. Additional unscheduled meetings will be called as needed with no less than 60 days notice.

DATA ACCESS

The guiding principle is that access to data generated by NEuroCOUGH CRC will be granted to all partners, including all the contributing funding partners. This access will allow partners to use the data for internal questions or evaluations. For new project proposals (see below) for which additional funding is provided by some of the funders only, access to these data will be initially limited to these funders, ultimately becoming available to all upon publication. Access to data for

publication purposes is set out in line with a specific Data Access and Publication process to ensure maximum benefit and to avoid overlap (see flow diagram below).

The NEuroCOUGH Registry Scientific Committee will have the primary role for screening applications for access to registry data and monitoring of progress with projects. They will be expected to act in accordance with the principles of the NEuroCOUGH registry protocol and to comply with agreements between contributing centres. In particular, the following principles should apply to decisions regarding data access

- Access to anonymised data through the “Safe Haven” platform will be possible for all Investigators and partner stakeholders throughout the study
- Active Investigators and other stakeholders will have unrestricted access to their own data.
- Request for data from industry or external agencies will be considered by the NEuroCOUGH Registry Scientific Committee but will incur a fee for service unless the parties have a prior agreement that supersedes this.
- Requests for data from individuals who do not contribute to the registry will be considered but may incur a fee for service at the discretion of the Registry Scientific Committee
- Identifiable patient data will never be released

Workflow for analysis of NEuroCOUGH Registry data

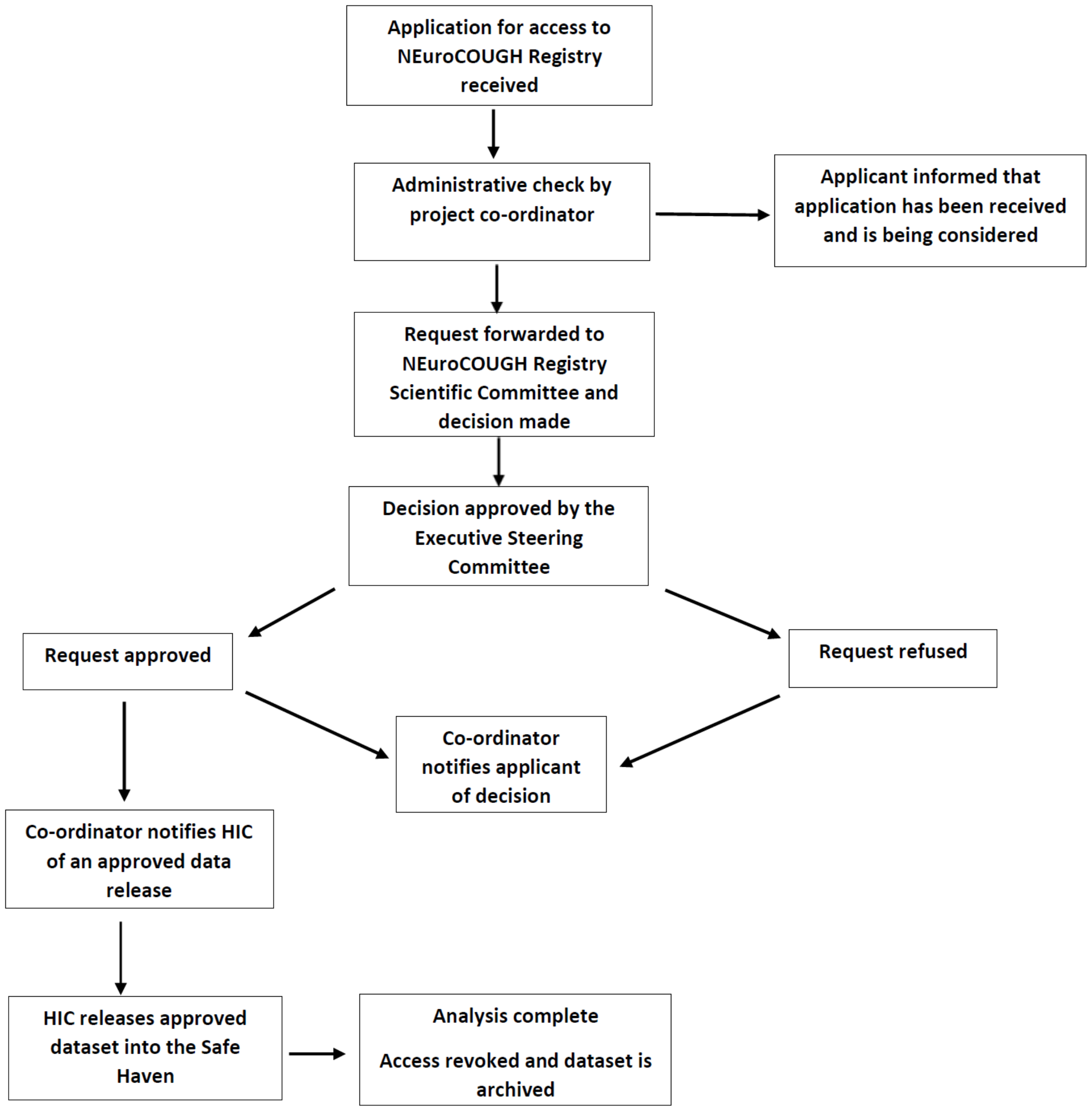

AUTHORSHIP AND PUBLICATIONS

It is anticipated that all clinical study reports arising from work undertaken by NEuroCOUGH investigators and partners will be used for publication and presentation at scientific meetings. Investigators have the right to publish orally or in writing the results of their study. However, the final decision to publish any aspect of the NEuroCOUGH study data rests with the Registry Scientific Committee. Investigators wishing to publish aspects of the study must submit a proposal for discussion and approval by the Registry Scientific Committee. NEuroCOUGH partners may ask to see

publications prior to submission and may request a reasonable delay in publication in order to protect intellectual property and/or to request removal of any confidential information belonging to the Sponsor, funder or collaborators.

The NEuroCOUGH CRC will follow the International Committee of Medical Journal Editors (ICMJE) recommendations regarding authorship. These are shown below for reference;

An author must take responsibility for at least one component of the work, should be able to identify who is responsible for each other component, and should ideally be confident in their co-authors' ability and integrity. Authorship credit should be based on 1) substantial contributions to conception and design, acquisition of data, or analysis and interpretation of data; 2) drafting the article or revising it critically for important intellectual content; and 3) final approval of the version to be published. Authors should meet conditions 1, 2, and 3.

When a large, multicentre group has conducted the work, the group should identify the individuals who accept direct responsibility for the manuscript. These individuals should fully meet the criteria for authorship/contributorship defined above, and editors will ask these individuals to complete journal-specific author and conflict-of-interest disclosure forms. When submitting a manuscript authored by a group, the corresponding author should clearly indicate the preferred citation and identify all individual authors as well as the group name. Journals generally list other members of the group in the Acknowledgments. The NLM indexes the group name and the names of individuals the group has identified as being directly responsible for the manuscript; it also lists the names of collaborators if they are listed in Acknowledgments.

It should be noted that;

- Acquisition of funding, collection of data, or general supervision of the research group alone does not constitute authorship.

- All persons designated as authors should qualify for authorship, and all those who qualify should be listed.

- Each author should have participated sufficiently in the work to take public responsibility for appropriate portions of the content.

All questions regarding publication or authorship will be addressed by the Registry Scientific Committee, who will have the final authority over these decisions.
